# CD8^+^ T cells mediate the antitumor activity of frankincense and myrrh in hepatocellular carcinoma

**DOI:** 10.1186/s12967-018-1508-5

**Published:** 2018-05-21

**Authors:** Chun Xu, Xian Lu, Wei Liu, Anxian Chen, Gang Meng, Hailin Zhang, Binghua Li, Yonghui Zhang, Junhua Wu, Jiwu Wei

**Affiliations:** 1 Jiangsu Key Laboratory of Molecular Medicine, Medical School of Nanjing University, Nanjing, 210093 China; 2 The Affiliated Drum Tower Hospital, Medical School of Nanjing University, Nanjing, 210093 China; 3 Nanjing University Hightech Institute at Suzhou, Suzhou, 215123 China

**Keywords:** Frankincense, Myrrh, Cancer-related inflammation, NF-κB/STAT3 signaling, Antitumor immunity, Hepatocellular carcinoma

## Abstract

**Background:**

Tumor-promoting inflammation is an emerging hallmark of cancer, which participates in both cancer progression and immune escape. Hepatocellular carcinoma (HCC) is a typical inflammation-related cancer with an extremely poor prognosis. Frankincense and myrrh are anti-inflammation agents commonly used in clinic. The purpose of this study is to investigate whether extract of frankincense and myrrh (FM) downregulates inflammatory microenvironment of HCC and thereby restores antitumor immune responses.

**Methods:**

The water-decocting FM was obtained and quantified. HCC cell lines HCCLM3 and Hepa1-6 were used to evaluate the efficacy of FM targeting NF-κB and STAT3 signaling with western blot and qRT-PCR analysis. CD8^+^NKG2D^+^ cells were derived from human peripheral blood and were used for evaluation of immune cells-mediated inflammation and oncolysis on HCCLM3 cells. The antitumor efficacy of FM was investigated both in immune compromised and immune competent mice bearing subcutaneous HCC. Mice received daily oral gavage of FM at 60 mg/kg. Immune activity within tumor microenvironment (TME) was assessed by ELISpot assay and flow cytometry, respectively. Depletion of CD8^+^ T cells or NK cells was achieved by intraperitoneal injection of respective neutralizing antibody.

**Results:**

FM significantly inhibited the activation of NF-κB and STAT3 signaling in HCC cells induced by cytokines (TNF-α or IL-6) and in co-culture system with CD8^+^NKG2D^+^ cells. Furthermore, FM sensitized HCC cells to CD8^+^NKG2D^+^ cells-mediated oncolysis. In HCC-bearing mice, FM at a non-toxic dose failed to reduce tumor growth in immune compromised mice, whereas it significantly inhibited tumor growth and prolonged life span in immune competent mice. While the number of IFN-γ-producing cells within TME was increased in mice treated with FM, the infiltration of CD8^+^ T cells and NK cells was not increased. Finally, we identified that depletion of CD8^+^ T cells rather than NK cells abrogated the antitumor activity of FM.

**Conclusions:**

Our results show for the first time that CD8^+^ T cells mediate the antitumor activity of FM at a non-toxic dose. This may provide new insights to this ancient mysterious prescription in cancer therapy, which offers a novel and practical therapeutic strategy and the possibilities of combined immunotherapy for HCC as well as other inflammation-related cancers in clinic.

## Background

Inflammation is regarded as one of the essential characteristics of cancer. It contributes to carcinogenesis, angiogenesis, invasion and metastasis [1]. It is estimated that 15–20% of cancers are associated with chronic infections and inflammation [2]. The typical examples of inflammation- and infection-associated cancers include colorectal cancer and inflammatory bowel diseases [3], gastric cancer and chronic *Helicobacter pylori* infection [4], and hepatocellular carcinoma (HCC) following chronic hepatitis virus HBV or HCV infection [5]. HCC accounts for 70–90% of liver cancers globally, which is estimated to be the second leading cause of cancer-related death [6].

In tumor and tumor microenvironment (TME), the transcription factors nuclear factor kappa-light-chain-enhancer of activated B-cells (NF-κB), and signal transducer and activator of transcription 3 (STAT3) are commonly constitutively activated, which results in an elevated level of inflammatory factors mediating tumor progression [7]. Tumor necrosis factor-α (TNF-α) is a major cytokine inducing NF-κB activation through IκB kinase (IKK)/NF-κB pathway [8]. Meanwhile, TNF-α is also a cytokine downstream of NF-κB. Although TNF-α has been shown to both inhibit and promote tumor growth, chronically produced TNF-α enhances tumor development in several cancer types [9]. Among the NF-κB target gene products, interleukin-6 (IL-6) is a key activator of STAT3. Activated STAT3 promotes expression of diverse immunosuppressive factors [10]. Accumulating studies show that anti-inflammatory therapeutics hold promise for cancer treatment. Epidemiological evidence strongly suggests that nonsteroidal anti-inflammatory drugs (NSAIDs), e.g. aspirin, could reduce cancer incidence. Other clinical or preclinical evidences also support that the anti-inflammatory agents targeting inflammatory cytokines and chemokines have the ability to inhibit cancer development [11].

Frankincense and myrrh are traditional herbal drugs against inflammation. Frankincense is the gum resin of species in the genus Boswellia of the family Burseraceae, while myrrh is the plant stem resinous exudate of species of Commiphora family [12]. Both of them have been used to treat inflammatory diseases and alleviate the pain or swelling of inflammation-related disorders since antiquity. Many pharmacological studies have investigated the mechanisms underlying the anti-inflammation function of frankincense and myrrh. Boswellic acid extracted from frankincense reduces NF-κB activation by inhibiting IKK mediated IκBα degradation [13]. Guggulsterone, a main functional extract of the myrrh, also inhibits the IKK/NF-κB pathway [14]. In addition, both boswellic acid and guggulsterone inhibit STAT3 activation through induction of a protein tyrosine phosphatase SHP-1 [15, 16]. In Chinese medicine, frankincense and myrrh are often combined to achieve a synergistic anti-inflammation effect [17].

In recent years, the compounds isolated from frankincense or myrrh, have been studied in cancer therapy. Due to the inhibitory activity of NF-κB or STAT3, boswellic acid analogue was shown to inhibit the growth and metastasis of human colorectal cancer in nude mice through downregulation of cancer proliferation, invasion, angiogenesis, etc. [18]. Guggulsterone was shown to inhibit tumor growth in various animal experimental models and to enhance the chemo-sensitivity of breast cancer cells [19, 20].

In infection-related HCC development, NF-κB and STAT3 are constitutively activated [21]. Given the critical role of chronic inflammation in tumor immunosuppression, and the potent anti-inflammatory effect of frankincense and myrrh, we sought to investigate the potency of extract of frankincense and myrrh (FM) in modulating antitumor immune responses in HCC models.

## Methods

### Cell lines

Human HCC cell line HCCLM3 was obtained from Live Cancer Institute of Zhongshan Hospital (Shanghai, China). Mouse HCC cell line Hepa1-6 was purchased from Shanghai Institutes for Biological Sciences, Chinese Academy of Science (Shanghai, China). Cells were cultured in Dulbecco’s modified Eagle’s medium (DMEM) supplemented with 10% fetal bovine serum (FBS), 100 units/ml penicillin and 100 μg/ml streptomycin (all from Life Technologies, USA). All cells were kept at 37 °C in a humidified atmosphere of 5% CO_2_ incubator.

### CD8^+^NKG2D^+^ cells

CD8^+^NKG2D^+^ cells were generated using our group’s protocol [22]. In brief, human peripheral blood lymphocytes were isolated from healthy volunteers. Cells were cultured in GT-T551 (SK551S, TaKaRa, Japan) medium supplemented with 2% autologous serum, 100 units/ml penicillin, 100 μg/ml streptomycin, 2000 IU/ml recombinant human IFN-γ (Chemo Wanbang Biopharma, Shanghai, China) for 24 h. To promote the expression of NKG2D, OK432 (Chugai Pharmaceutical, Chome, Japan), an immunotherapeutic agent prepared from inactivated Streptococcus pyogenes, was added into the culture medium at the concentration of 10 mg/ml [23]. On 2nd day, cells were transferred to a flask pre-coated with anti-CD3 antibody (T&L Biological Technology, Beijing, China). GT-T551 medium supplemented with 700 IU/ml recombinant human IL-2 (BD Biosciences, USA) was used for further culture. After 2 weeks, cells were subjected to flow cytometry to control the phenotype and quality.

### Reagents

Frankincense (#71202000) and myrrh (#71202500) were purchased from Jiangsu Province Hospital of Traditional Chinese Medicine (Nanjing, China). Recombinant human TNF-α (HNAE-10620), mouse TNF-α (MNAE-50349), human IL-6 (HNAE-10395), and mouse IL-6 (MNAE-50136) were all obtained from Sino Biotechnology (Beijing, China).

### Water-decocting extract of frankincense and myrrh

The extract of frankincense and myrrh was prepared as previously described [24]. Briefly, powered resin of frankincense (1.0 kg) or myrrh (1.0 kg) was extracted with water (2 × 10 L) under reflux for 1 h. The extraction was repeated twice. Then the extracted solution was boiled in a reflux condensation device for 2 h, cooled to room temperature (RT), and centrifuged at 3000 rpm for 15 min to remove the residue. Finally, the supernatant was evaporated in a rotary evaporator for 12 h to obtain the powder of frankincense or myrrh.

To prepare the solution, 100 mg of frankincense powder and 100 mg of myrrh powder were dissolved in 5 ml cell culture medium (for in vitro experiments) or PBS (for in vivo therapy) in water bath at 90 °C overnight. Then the supernatant was obtained after centrifugation at 12,000 rpm for 30 min followed by filtration through a 0.22 μm filter.

### 3-(4,5-Dimethylthiazol-2-yl)-2,5-diphenyltetrazolium bromide (MTT) assay

MTT (M5655, Sigma, USA) (5 mg/ml, 20 μl) was added into each well in 96-well plate and incubated for 3 h at 37 °C. Then, the supernatant was discarded. 100 μl of isopropyl alcohol (12090611516, Nanjing Chemical Reagent Co., China) was then added and agitated for 20 min to dissolve the crystallization. The absorbance was measured using a Multimode Reader (SMP500-13497-JWYK, Molecular Devices, USA) at 570 nm. Cell viability was calculated as the ratio of the absorbance of treated cells to that of controls.

### Trypan blue exclusion test

Harvested cells were stained with 0.2% trypan blue (C3601-2, Beyotime, China). Cell numbers and viability were determined with a Countstar Automated cell counter (Inno-Alliance Biotech, USA).

### Luciferase assay

To test the oncolytic effect of CD8^+^NKG2D^+^ cells, HCCLM3 cells stably expressing luciferase (HCCLM3-luciferase) were used as target cell. The viability of HCCLM3-luciferase cells was determined by luciferase assay according to manufacturer’s instructions (E1500, Promega, USA). After co-culture in a black 96-well plate, cell lysis buffer was added into each well for 15 min. Luciferase assay substrate was added and then the plate was subjected to GloMax 96 microplate luminometer (Promega, USA).

### Western blot analysis

Cells were lysed in radio-immunoprecipitation assay buffer containing a protease inhibitor cocktail (11873580001, Roche, Switzerland). The protein concentration was determined using BCA Kit (P10010, Beyotime, China). Equal amounts of protein were separated by SDS-PAGE and electrophoretically transferred onto polyvinylidene difluoride membranes (03010040001, Roche, Switzerland). The membranes were blocked, incubated with the specific primary antibodies, washed, and followed with incubation with appropriate horseradish peroxidase-conjugated secondary antibodies. Signals were detected using an enhanced chemiluminescence reagent (WBKLS0500, Millipore, USA) and subjected to the Alpha Innotech Fluor Chem-FC2 imaging system (ProteinSimple, USA). The gray value was analyzed with Software Image J (freely available at https://imagej.nih.gov/ij/download.html). Antibodies used were as follows: anti-glyceraldehyde-3-phosphate dehydrogenase (anti-GAPDH) (MB001, Bioworld, USA; 1:5000 diluted), anti-IκBα (4814S, Cell Signaling Technology, USA, 1:1000 diluted), anti-phospho-IκBα (2859S, Cell Signaling Technology, USA; 1:1000 diluted), anti-STAT3 (9139S, Cell Signaling Technology, USA; 1:1000 diluted), and anti-phospho-STAT3 (9145S, Cell Signaling Technology, USA; 1:1000 diluted).

### Quantitative RT-PCR analysis of mRNA expression

Total cellular RNA was extracted with TRIzol (15596-026, Thermo Scientific, USA), and was reverse-transcribed using the synthesis system (RR036A, TaKaRa, Japan). Then SYBR green PCR master mix (04913914001, Roche, Switzerland) reagent was used according to the manufacturer’s protocol and the PCR was performed using the Real-Time PCR system (Viia7, Thermo Scientific, USA). Gene expression was calculated with the comparative threshold cycle (*C*_*T*_) method and normalized to the endogenous levels of GAPDH. Primer sequences used were as follows:

human *GAPDH* forward 5′- CCATGTTCGTCATGGGTGTGAACCA-3′ and reverse 5′-GCCAGTAGAGGCAGGGTGATGTTC-3′;

human *TNF*-*α* forward 5′- CCCAGGGACCTCTCTCTCTAATC-3′ and reverse 5′- ATGGGCTACAGGCTTGTCACT-3′;

human *IL*-*6* forward 5′- GGTACATCCTCGACGGCATCT-3′ and reverse 5′- GTGCCTCTTTGCTGCTTTCAC-3′;

mouse *gapdh* forward 5′-TCTCCTGCGACTTCAACA-3′ and reverse 5′- TGTAGCCGTATTCATTGTCA -3′;

mouse *tnf*-*α* forward 5′- ACGCTCTTCTGTCTACTGA -3′ and reverse 5′- GCCATAGAACTGATGAGAGG-3′;

mouse *il*-*6* forward 5′- CCACCAAGAACGATAGTCA -3′ and reverse 5′- TTGTCACCAGCATCAGTC -3′.

### Mice, tumor engraftment, and in vivo experiments

4–6-week-old male C57BL/6 mice and Balb/c nude mice were obtained from Nanjing Biomedical Research Institute of Nanjing University (Nanjing, China). Mice were kept in the conditional microenvironment (temperature 22 ± 2 °C, humidity 60 ± 10%, 12/12 h light/dark cycle).

To establish HCC xenograft models, each mouse was subcutaneously injected with 5 × 10^6^ Hepa1-6 cells or 1 × 10^7^ HCCLM3 cells suspended in 200 μl PBS containing 10% Matrigel Matrix (354234, Corning, USA). Therapy started on day 7 post tumor cell inoculation, when mice showed visible tumor nodules (4–6 mm in diameter). FM was given daily by oral gavage at 60 mg/kg until the therapy stopped at the indicated time point.

For the CD8 and NK cells depletion experiment, 500 μg of anti-CD8α mAb (clone YTS 169.4, BE0017, BioXCell, USA) or anti-NK mAb (clone PK136, BE0036, BioXCell, USA) was injected intraperitoneally every 2 weeks from 1 day before therapy. Depletion of CD8 or NK cells was confirmed by fluorescence-activated cell sorting (FACS) analysis of peripheral blood.

For all the in vivo experiments, tumor volume was monitored every 2–3 days by caliper measurement and calculated by length × width^2^/2. Mice were euthanized when tumor volume reached 2 cm^3^, or when mice appeared moribund.

### Flow cytometry

For CD8^+^NKG2D^+^ cells analysis, cells were harvested and stained with anti-CD3-FITC (E10472-1633, eBioscience, USA), anti-CD8-PE (561946, BD, USA), anti-NKG2D-APC (130-099-216, Miltenyi Biotec, Germany), anti-CD27-PE (560985, BD, USA), and anti-CD57-APC (560845, BD, USA) antibodies. To detect the infiltration of CD8 or NK cells in the tumor mass, tumors were removed and crushed into single cell suspension. Then cells were stained with mouse anti-CD8-PerCp (46-0083-80, eBioscience, USA) or anti-NK1.1-FITC (553164, BD, USA) antibodies. To confirm the depletion of CD8 or NK cells, cells were isolated from peripheral blood and stained with anti-CD8-PerCp (46-0083-80) or anti-NK1.1-PerCp antibodies (45-5941-82) (all from eBioscience, USA). The staining was performed on ice for 30 min. Samples were acquired on the FACS Calibur (BD, USA) and data was analyzed using FlowJo software (Version 8.5.3, Tree Star Inc., USA).

### IFN-γ enzyme-linked immunosorbent spot (ELISpot) assay

Immunity activity in the TME was measured with mouse IFN-γ ELISpot Kit (3321-2A, Mabtech, Sweden) according to the guidelines. In brief, the plate was coated with mouse INF-γ capture antibodies and kept overnight at 4 °C in the dark. Cells isolated from tumor masses were seeded at a density of 1.5 × 10^5^ cells/well. After 24 h incubation at 37 °C in a humidified atmosphere of 5% CO_2_ incubator, cells were removed and the wells were rinsed with PBS. The biotinylated detection antibodies against IFN-γ were then added and the plate was incubated for 2 h at RT. Then the wells were rinsed with PBS followed by incubation with Streptoavidin-ALP for 1 h. The substrate BCIP/NBT-plus was then added. The reaction was stopped by washing wells with tap water when spots emerged. The plate was subjected to ELISpot reader system (Autoimmun Diagnostika GmbH, Germany) to detect spots number and activity. The activity of spot was characterized as the average of spot size and intensity for each well.

### Statistical analysis

The results are presented as mean ± SD. Statistical analyses were performed using Student’s t test. Animal survival is presented using Kaplan–Meier survival curves and was statistically analyzed using log rank test (GraphPad Prism version 6). p value < 0.05 was considered to be statistically significant.

## Results

### FM efficiently reduces the activation of NF-κB and STAT3 in HCC cells

**Fig. 1:**
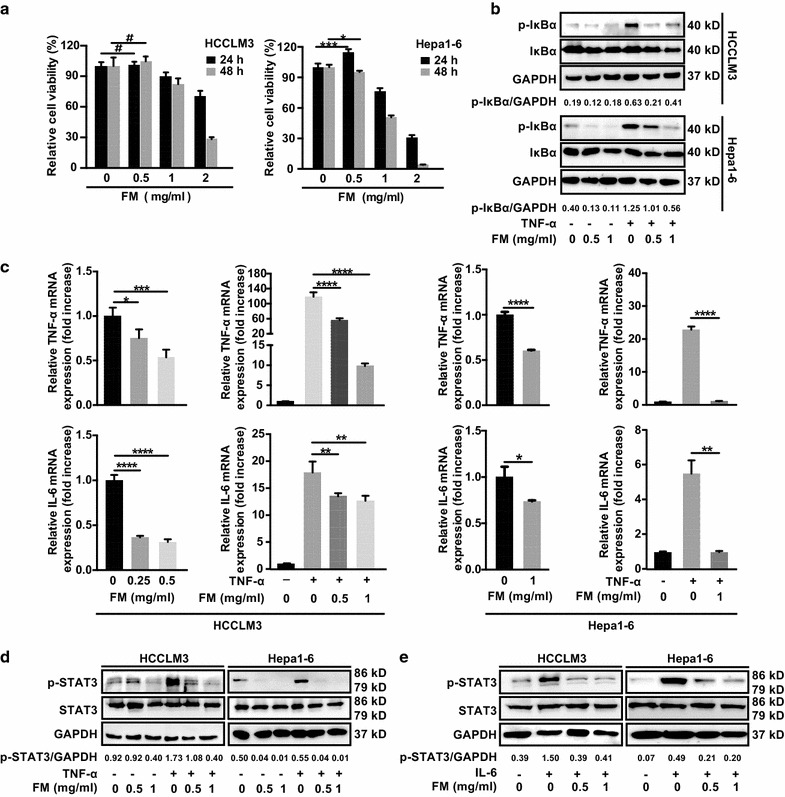
FM reduces the activation of NF-κB and STAT3 in HCC cells. **a** Human HCCLM3 or mouse Hepa1-6 HCC cells were seeded in 96-well plates overnight. Then cells were treated with serial concentrations of FM. 24 and 48 h later, cells were subjected to MTT assay. **b**–**e** 2 × 10^5^ HCCLM3 or Hepa1-6 cells were seeded into 12-well plates. Cells were treated with FM at indicated concentrations for 24 h and then stimulated with recombinant TNF-α (human, 15 ng/μl; mouse, 80 ng/μl) or recombinant IL-6 (both at 25 ng/μl). Total protein or mRNA was extracted and subjected to western blot or qRT-PCR analysis. **b** The protein level of total/phosphorylated IκBα, **c** mRNA level of TNF-α and IL-6, and **d** protein level of total/phosphorylated STAT3 after TNF-α stimulation are shown. GAPDH was used as loading control. **e** Phosphorylation of STAT3 after IL-6 stimulation is shown. The results of qRT-PCR are shown as Mean ± SD. Similar results were obtained in three independent experiments. ^#^p > 0.05, *p < 0.05, **p < 0.01, ***p < 0.001, ****p < 0.0001

To investigate the anti-inflammatory activity of FM, cytotoxicity assay of FM on HCC cells was firstly conducted to obtain non-toxic concentrations (Fig. 1a). We found that FM strongly inhibited the phosphorylation of IκBα both in the absence or presence of TNF-α in a dose-dependent manner (Fig. 1b). Of note, at a non-toxic concentration of 0.5 mg/ml, FM exerted a significant reduction of p-IκBα. In line, the expression of NF-κB-regulated genes including TNF-α and IL-6 was significantly decreased in HCC cells treated with FM in the absence or presence of TNF-α stimulation (Fig. 1c). Meanwhile, the level of phosphorylated STAT3 was also dramatically reduced (Fig. 1d). Moreover, FM also massively inhibited the phosphorylation of STAT3 stimulated by IL-6 in HCC cells (Fig. 1e). Therefore, our data confirm that FM possesses a potent inhibitory effect on NF-κB and STAT3 activation.

### FM mitigates NF-κB/STAT3 activation triggered by CD8^+^NKG2D^+^ cells -and enhances oncolytic efficacy of CD8^+^NKG2D^+^ cells on HCC cells

**Fig. 2:**
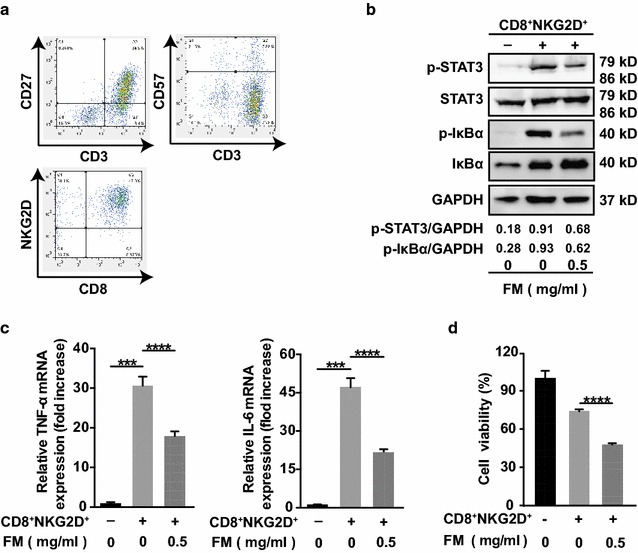
FM mitigates CD8^+^NKG2D^+^ cell-activated inflammatory signaling and enhances the oncolysis of CD8^+^NKG2D^+^ cells. **a** CD8^+^NKG2D^+^ cells were generated as described in methods. Cells were identified by flow cytometry with anti-CD3, anti-CD8, anti-NKG2D, anti-CD27 and anti-CD57 antibodies. **b**–**d** HCCLM3 cells stably expressing luciferase (HCCLM3-luciferase) (target cells, T) were pre-treated with FM at a non-toxic dose of 0.5 mg/ml for 12 h or left untreated, then medium was discarded. CD8^+^NKG2D^+^ cells (effector cells, E) were added at a ratio of 5:1 (E: T) in fresh medium for 12 h. Then, cells were harvested for western blot, qRT-PCR analysis, or luciferase activity measurement. **b** The protein level of IκBα, phosphorylated IκBα, STAT3 and phosphorylated STAT3, **c** mRNA level of TNF-α (left panel) and IL-6 (right panel), and **d** luciferase activity reflecting the cell viability are shown. GAPDH was used as loading control. Similar results were obtained in two independent experiments. Mean ± SD of each group are shown. ***p < 0.001, ****p < 0.0001

Next, we wanted to know whether reduced activation of NF-κB and STAT3 could result in an improved antitumor immunity. To this end, CD8^+^NKG2D^+^ cells were employed due to their NKT character. CD8^+^NKG2D^+^ cells were generated from human peripheral blood and could be activated directly by their cognate ligands such as MICA/B often expressed on the surface of tumor cells. Therefore, no antigen-presenting cells were needed for the activation of T cells in the in vitro settings. Previous study found that naïve-derived effector CD8^+^ T cells, which expressed higher CD27 and lower CD57, exhibited higher proliferative potential with better therapeutic efficacy in clinic [25]. Hence, the differentiation status of CD3^+^ T cells was firstly qualified by CD27 or CD57 antibodies staining. Next, we determined the frequency of CD8 and NKG2D double positive cells gated from CD3^+^ cells (Fig. 2a). At a non-toxic dose (0.5 mg/ml), FM markedly reduced the activation of NF-κB and STAT3 in the mixture of HCC and CD8^+^NKG2D^+^ cells (Fig. 2b), and downregulated the expression of TNF-α and IL-6 (Fig. 2c). We then further determined the oncolysis of CD8^+^NKG2D^+^ cells on HCC cells pre-treated with or without FM. We found that FM pre-treated HCC cells were more sensitive to CD8^+^NKG2D^+^ cell-mediated oncolysis (Fig. 2d). These results show that FM reduces immune cells-triggered inflammation and enhances the sensitivity of HCC cells to the cytotoxicity of immune cells.

### An intact immune system is indispensable to the antitumor efficacy of FM at a non-toxic dose

**Fig. 3:**
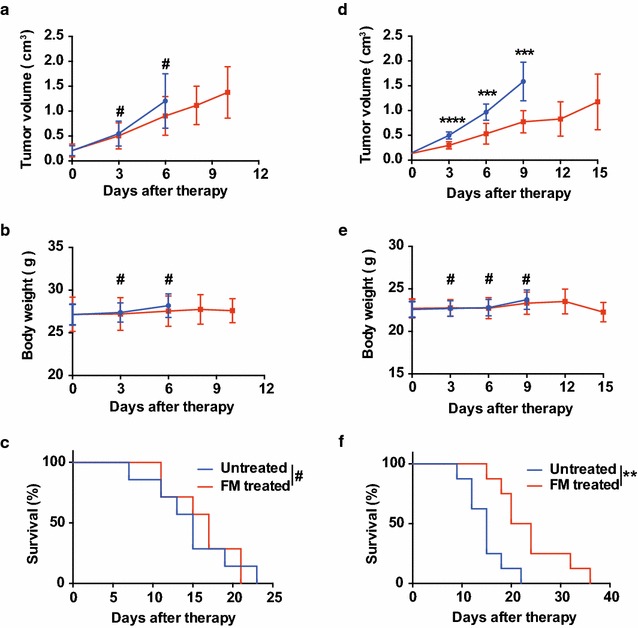
An intact immune system is indispensable to the antitumor efficacy of FM. 4–6-week-old male (**a**) Balb/c nude mice received subcutaneous injection of 1 × 10^7^ HCCLM3 cells, or (**d**) C57BL/6 mice received 5 × 10^6^ Hepa1-6 cells in the right flank. When tumor size reached about 4–6 mm in diameter, mice were randomly divided into two groups. Then the mice were either treated daily with oral gavage of FM at a dose of 60 mg/kg, or left untreated as a control (n = 8 in each group). Tumor volumes (**a**, **d**) were measured by caliper and body weight (**b**, **e**) were monitored every 2–3 days during the treatment. Mean ± SD of each group are shown. Mice were sacrificed when tumor volume reached 2 cm^3^, or when mice appeared moribund. **c**, **f** Survival was determined and plotted for Kaplan–Meier survival analysis and analyzed by log-rank test. ^#^p > 0.05, **p < 0.01, ***p < 0.001, ****p < 0.0001

It has been shown that the purified extract of frankincense (boswellic acid), essential oil of frankincense, and extract of myrrh (guggulsterone) exhibit antitumor capabilities in immunocompromised mice [18, 26, 27]. However, it is not yet clear if the water-decocting FM, which is commonly used in clinic, also has the similar antitumor efficacy. Then we performed an in vivo experiment in nude mice bearing subcutaneous human HCC. The mice were treated with FM at a dose which previously has been shown to inhibit inflammatory responses in an arthritis rat model [17]. To our surprise, FM failed to inhibit tumor growth, and there was no difference of survival time between treated and untreated groups (Fig. 3a, c). Next, we wanted to know the antitumor efficiency of FM in immune competent mice. Therefore, we performed another in vivo experiment in HCC-bearing mice with intact immunity using the same dose of FM. Indeed, FM significantly inhibited tumor growth and prolonged lifespan of mice from an average of 15 d in control group to an average of 22 d in FM treated group (Fig. 3d, f). In both tumor models, FM had no obvious influence on the body weight (Fig. 3b, e). These data suggest that the water-decocting FM at the anti-inflammatory dose (non-toxic dose) also possesses an antitumor ability, which depends on the host immunity system.

### FM sensitizes HCC to the cytotoxicity of immune cells in the TME

**Fig. 4:**
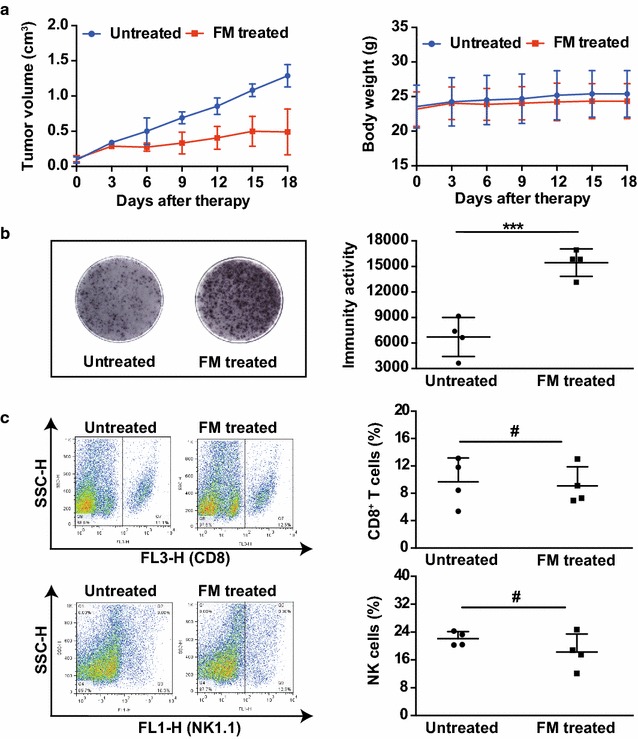
FM increases amount of antitumor cytokine IFN-γ within TME. C57BL/6 mice bearing subcutaneous HCC received oral gavage of FM (60 mg/kg/day, n = 4) or left untreated (n = 4) 0.18 days later, tumors were dissected. Single cell suspensions were obtained from tumor tissue and cells were counted after trypan blue staining using CountStar. **a** Tumor volumes and body weight were monitored every 3 days. Mean ± SD of each group are shown. **b** Immune activity in the TME was detected by the mouse IFN-γ ELISpot assay kit (left panel) and means of immune activity in each group are shown (right panel). **c** Single cell suspensions were stained with anti-CD8-PerCp or anti-NK1.1-FITC antibodies and then subjected to flow cytometry to determine the percentage of CD8^+^ T cells (upper panel) or NK cells (lower panel). Representative flow plots (left panel) and mean ± SD of each group (right panel) are shown. ^#^p > 0.05, ***p < 0.001

Having shown that the antitumor capability of FM is immune-dependent, we next investigated if the immune activity in the TME could be modulated by FM. In the immune competent C57BL/6 mice, we established a subcutaneous murine HCC model and monitored the tumor growth and body weight during the intervention (Fig. 4a). Then, using ELISpot assay, we found that the amount of antitumor cytokine IFN-γ was robustly upregulated within the TME from the mice treated with oral garage of FM (Fig. 4b). Then we asked if this effect was contributed by increased infiltration of immune cells. However, by determination of CD8^+^ T and NK cells from the cell suspensions isolated from tumor masses, we did not observe the increased infiltration of either CD8^+^ T (upper panel) or NK cells (lower panel) in FM treated mice (Fig. 4c). The results indicate that FM may restore immune responses against HCC in the TME.

### CD8^+^ T cells mediate the antitumor activity of FM

**Fig. 5:**
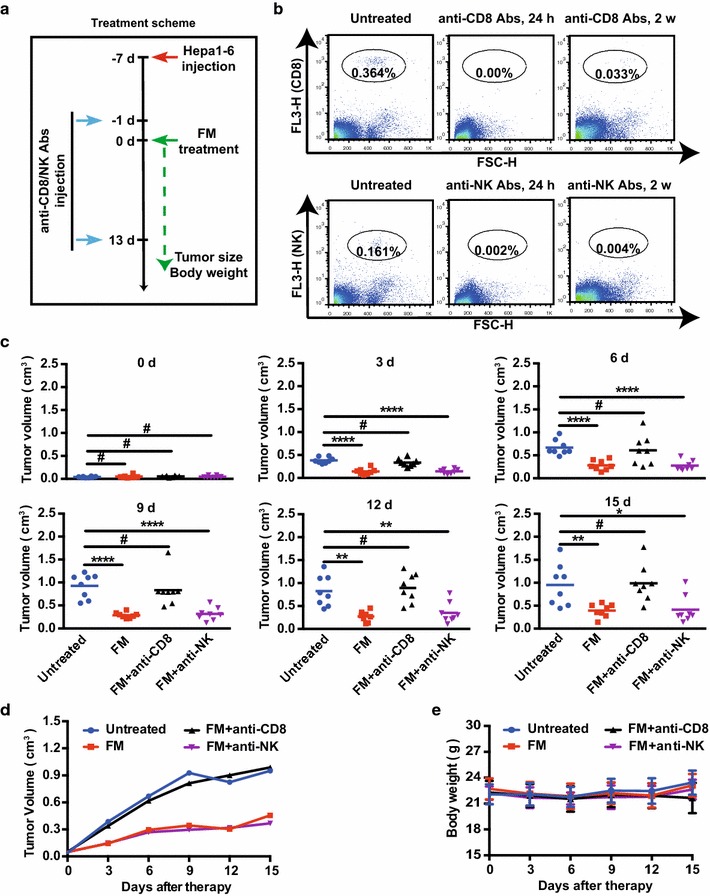
CD8^+^ T cells mediate antitumor activity of FM in vivo. 4- to 6-week-old male C57BL/6 mice received subcutaneous injection of 5 × 10^6^ Hepa1-6 cells. When tumor volumes reached about 4–6 mm in diameter, mice were randomized to four groups. Anti-CD8 or anti-NK neutralizing antibodies were injected intraperitoneally (500 μg/mouse) 1 day prior to and 13 days after FM treatment (60 mg/kg/day, n = 8). Mice in other two groups received oral gavage of FM alone (60 mg/kg/day, n = 8), or left untreated (n = 8). **a** The scheme depicts the schedules of the interventions. **b** Peripheral blood was obtained from each mouse, stained with anti-CD8-PerCp or anti-NK1.1-PerCp antibodies and then subjected to flow cytometry to monitor the depletion of CD8 or NK cells 1 and 13 days after first injection of neutralizing antibodies. **c**–**e** Tumor volume and body weight were monitored every 3 days during the treatment. **c** Tumor volume of each mouse in each group is shown at each monitoring time point. **d** An overview of tumor growth **e** and body weight are also shown. Mean ± SD of each group are shown. ^#^p > 0.05, *p < 0.05, **p < 0.01, ****p < 0.0001

Next, we wanted to figure out which subset of lymphocytes contributed to the antitumor activity of FM. In immune competent mice bearing HCC, we depleted either CD8^+^ T cells by anti-CD8 or NK cells by anti-NK neutralizing antibodies 1 day before FM treatment (Fig. 5a). The depletion efficacy was monitored 1 and 13 days after administration of antibodies by flow cytometry (Fig. 5b). Then we monitored the tumor growth, and found that the antitumor effect of FM was completely abrogated by the depletion of CD8^+^ T cells, but not by the depletion of NK cells (Fig. 5c, d). No side effects were observed during the experiment as monitored by body weight (Fig. 5e). In conclusion, our results illustrate CD8^+^ T cells mediate the antitumor activity of FM.

## Discussion

HCC is a type of inflammation-related cancer. In this study, we showed that the water-decocting FM effectively targeted NF-κB/STAT3 signaling in HCC at a non-toxic dose, and increased release of antitumor cytokine IFN-γ in TME. Further, we identified that CD8^+^ T cells mediated the antitumor activity of FM. Our results show for the first time a novel antitumor function/mechanism of this ancient anti-inflammation prescription, which provides a potential treatment strategy in clinical cancer therapy.

The extract of frankincense or myrrh has shown preliminary ability to inhibit cancer in vitro and several in vivo studies. Most of the studies focused on elucidating the anti-cancer mechanisms such as apoptosis induction, angiogenesis inhibition, drug resistance reduction by targeting NF-κB and/or STAT3 [18, 27–29]. Beyond this, as a traditional Chinese medicine, the *Xihuang* pill composed of frankincense, myrrh, moschus and calculus bovis has been used for cancer therapy since the eighteenth century [30]. Nevertheless, how FM modulates the immunity against cancer is unclear.

To clarify the role of FM in antitumor immunity, the dose of FM used in this study was non-toxic to HCC cells in vitro, and also was non-toxic in a HCC-bearing immunocompromised mouse model, in which no significant side effect was observed between FM-treated and untreated mice. However, using the same dose, FM markedly reduced the activation of NF-κB and STAT3, significantly enhanced CD8^+^NKG2D^+^ cell-mediated antitumor efficacy in vitro, and exerted an effective therapeutic potency in HCC-bearing mice with intact immune system. Thus, FM at a non-toxic dose might be able to modulate immune responses against HCC.

Our results seem contradictory with some previous studies showing that the extract of frankincense or myrrh inhibits tumor growth in T cell deficient nude mice. The plausible explanation is that the given doses and components were different. For instance, the FM used in this study was water-decocting extract at a dose of 60 mg/kg/day, while the agent used in other studies was the identified and purified effective component such as boswellic acid at 100 mg/kg/day [18] or guggulsterone at 40–250 mg/kg/day [26, 31]. It would be interesting to investigate if the latter two components also improve antitumor immunity at relative lower non-toxic doses.

The efficacy of immunotherapy for solid tumors relies on the infiltration and the functional activity of immune cells in the TME [32]. Another novel finding in this study is that CD8^+^ T cells rather than the NK cells, mediated antitumor activity of FM. We found that the infiltration of CD8^+^ T cells was not increased in FM-treated mice, indicating that the enhanced release of IFN-γ might be contributed by the increased activity of CD8^+^ T cells within TME. The restored activity of CD8^+^ T cells should be a result of reducing the immunosuppressive microenvironment of HCC by FM. Previous studies showed that TNF-α induced apoptosis of mature T cells and CD8^+^ T cells [33, 34]. FM downregulated TNF-α/NF-κB activation in HCC, which may prevent CD8^+^ T cells undergoing apoptosis. It has also been shown that STAT3 activation in tumor participated in immune suppression on CD8^+^ T cells [35–37], and thus, FM may restore the activity of CD8^+^ T cells by inhibiting STAT3 activation. Taken together, since NF-κB and/or STAT3 are constitutively activated in HCC [21], FM may restore the otherwise suppressed activity of CD8^+^ T cells within TME by targeting NF-κB/STAT3 signaling.

## Conclusions

Our study sheds light on the importance of anti-inflammatory intervention in immunotherapy for inflammation-related cancers such as HCC. As the water-decocting FM is the commonly used formula in clinics and is also economic, our finding offers a novel and practical strategy for HCC immunotherapy considering the cancer-related inflammation.

## Abbreviations

FM: extract of frankincense and myrrh
HCC: hepatocellular carcinoma
IKK: IκB kinase
IL-6: interleukin-6
NF-κB: nuclear factor kappa-light-chain-enhancer of activated B-cells
STAT3: signal transducer and activator of transcription 3
TME: tumor microenvironment
TNF-α: tumor necrosis factor-α

## References

[1] Hanahan D, Weinberg RA (2011). Hallmarks of cancer: the next generation. Cell.

[2] Kuper H, Adami HO, Trichopoulos D (2000). Infections as a major preventable cause of human cancer. J Intern Med.

[3] Kulaylat MN, Dayton MT (2010). Ulcerative colitis and cancer. J Surg Oncol.

[4] Suerbaum S, Michetti P (2002). Helicobacter pylori Infection. New Engl J Med.

[5] Seeff LB (2004). Introduction: the burden of hepatocellular carcinoma. Gastroenterology.

[6] Torre LA, Siegel RL, Ward EM, Jemal A (2016). Global cancer incidence and mortality rates and trends—an update. Cancer Epidem Biomar.

[7] Multhoff G, Molls M, Radons J (2011). Chronic inflammation in cancer development. Front Immunol.

[8] Ghosh S, Karin M (2002). Missing pieces in the NF-kappaB puzzle. Cell.

[9] Balkwill F (2009). Tumour necrosis factor and cancer. Nat Rev Cancer.

[10] Kortylewski M, Yu H (2008). Role of Stat3 in suppressing anti-tumor immunity. Curr Opin Immunol.

[11] Crusz SM, Balkwill FR (2015). Inflammation and cancer: advances and new agents. Nat Rev Clin Oncol.

[12] El Ashry ES, Rashed N, Salama OM, Saleh A (2003). Components, therapeutic value and uses of myrrh. Pharmazie.

[13] Syrovets T, Büchele B, Krauss C, Laumonnier Y, Simmet T (2004). Acetyl-boswellic acids inhibit lipopolysaccharide-mediated TNF-α induction in monocytes by direct interaction with IκB kinases. J Immunol.

[14] Shishodia S, Aggarwal BB (2004). Guggulsterone inhibits NF-kappaB and IkappaBalpha kinase activation, suppresses expression of anti-apoptotic gene products, and enhances apoptosis. J Biol Chem.

[15] Ahn KS, Sethi G, Sung B, Goel A, Ralhan R, Aggarwal BB (2008). Guggulsterone, a farnesoid X receptor antagonist, inhibits constitutive and inducible STAT3 activation through induction of a protein tyrosine phosphatase SHP-1. Cancer Res.

[16] Kunnumakkara AB, Nair AS, Sung B, Pandey MK, Aggarwal BB (2009). Boswellic acid blocks signal transducers and activators of transcription 3 signaling, proliferation, and survival of multiple myeloma via the protein tyrosine phosphatase SHP-1. Mol Cancer Res.

[17] Su S, Duan J, Chen T, Huang X, Shang E, Yu L, Wei K, Zhu Y, Guo J, Guo S (2015). Frankincense and myrrh suppress inflammation via regulation of the metabolic profiling and the MAPK signaling pathway. Sci Rep.

[18] Yadav VR, Prasad S, Sung B, Gelovani JG, Guha S, Krishnan S, Aggarwal BB (2012). Boswellic acid inhibits growth and metastasis of human colorectal cancer in orthotopic mouse model by downregulating inflammatory, proliferative, invasive and angiogenic biomarkers. Int J Cancer.

[19] Bhat AA, Prabhu KS, Kuttikrishnan S, Krishnankutty R, Babu J, Mohammad RM, Uddin S (2017). Potential therapeutic targets of guggulsterone in cancer. Nutr Metab (Lond).

[20] Xu HB, Shen ZL, Fu J, Xu LZ (2014). Reversal of doxorubicin resistance by guggulsterone of *Commiphora mukul* in vivo. Phytomedicine.

[21] He G, Karin M (2011). NF-kappaB and STAT3—key players in liver inflammation and cancer. Cell Res.

[22] Chen A, Zhang Y, Meng G, Jiang D, Zhang H, Zheng M, Xia M, Jiang A, Wu J, Beltinger C, Wei J (2017). Oncolytic measles virus enhances antitumour responses of adoptive CD8(+) NKG2D(+) cells in hepatocellular carcinoma treatment. Sci Rep.

[23] Sakamoto N, Ishikawa T, Kokura S, Okayama T, Oka K, Ideno M, Sakai F, Kato A, Tanabe M, Enoki T (2015). Phase I clinical trial of autologous NK cell therapy using novel expansion method in patients with advanced digestive cancer. J Transl Med.

[24] Su S, Hua Y, Wang Y, Gu W, Zhou W, Duan JA, Jiang H, Chen T, Tang Y (2012). Evaluation of the anti-inflammatory and analgesic properties of individual and combined extracts from *Commiphora myrrha*, and *Boswellia carterii*. J Ethnopharmacol.

[25] Hinrichs CS, Borman ZA, Gattinoni L, Yu Z, Burns WR, Huang J, Klebanoff CA, Johnson LA, Kerkar SP, Yang S (2011). Human effector CD8(+) T cells derived from naive rather than memory subsets possess superior traits for adoptive immunotherapy. Blood.

[26] An MJ, Cheon JH, Kim SW, Kim ES, Kim TI, Kim WH (2009). Guggulsterone induces apoptosis in colon cancer cells and inhibits tumor growth in murine colorectal cancer xenografts. Cancer Lett.

[27] Ni X, Suhail MM, Yang Q, Cao A, Fung KM, Postier RG, Woolley C, Young G, Zhang J, Lin HK (2012). Frankincense essential oil prepared from hydrodistillation of Boswellia sacra gum resins induces human pancreatic cancer cell death in cultures and in a xenograft murine model. BMC Complem Altern M.

[28] Park B, Prasad S, Yadav V, Sung B, Aggarwal BB (2011). Boswellic acid suppresses growth and metastasis of human pancreatic tumors in an orthotopic nude mouse model through modulation of multiple targets. PLoS ONE.

[29] Renata M, Ewa I (2010). Chemopreventive and chemotherapeutic effect of trans-resveratrol and its analogues in cancer. Polski Merkuriusz Lekarski.

[30] Guo Q, Lin J, Liu R, Gao Y, He S, Xu X, Hua B, Li C, Hou W, Zheng H, Bao Y (2015). Review on the applications and molecular mechanisms of Xihuang pill in tumor treatment. Evid-Based Compl Alt.

[31] Sarfaraz S, Siddiqui IA, Syed DN, Afaq F, Mukhtar H (2008). Guggulsterone modulates MAPK and NF-κB pathways and inhibits skin tumorigenesis in SENCAR mice. Carcinogenesis.

[32] Fridman WH, Pages F, Sautes-Fridman C, Galon J (2012). The immune contexture in human tumours: impact on clinical outcome. Nat Rev Cancer.

[33] Zheng L, Fisher G, Miller RE, Peschon J, Lynch DH, Lenardo MJ (1995). Induction of apoptosis in mature T cells by tumour necrosis factor. Nature.

[34] Bertrand F, Rochotte J, Colacios C, Montfort A, Andrieu-Abadie N, Levade T, Benoist H, Ségui B (2016). Targeting TNF alpha as a novel strategy to enhance CD8(+) T cell-dependent immune response in melanoma?. Oncoimmunology.

[35] Yu H, Kortylewski M, Pardoll D (2007). Crosstalk between cancer and immune cells: role of STAT3 in the tumour microenvironment. Nat Rev Immunol.

[36] Kortylewski M, Kujawski M, Wang T, Wei S, Zhang S, Pilon-Thomas S, Niu G, Kay H, Mule J, Kerr WG (2005). Inhibiting Stat3 signaling in the hematopoietic system elicits multicomponent antitumor immunity. Nat Med.

[37] Kong LY, Wei J, Sharma AK, Barr J, Abou-Ghazal MK, Fokt I, Weinberg J, Rao G, Grimm E, Priebe W, Heimberger AB (2009). A novel phosphorylated STAT3 inhibitor enhances T cell cytotoxicity against melanoma through inhibition of regulatory T cells. Cancer Immunol Immun.

